# BTK gatekeeper residue variation combined with cysteine 481 substitution causes super-resistance to irreversible inhibitors acalabrutinib, ibrutinib and zanubrutinib

**DOI:** 10.1038/s41375-021-01123-6

**Published:** 2021-02-01

**Authors:** H. Yesid Estupiñán, Qing Wang, Anna Berglöf, Gerard C. P. Schaafsma, Yuye Shi, Litao Zhou, Dara K. Mohammad, Liang Yu, Mauno Vihinen, Rula Zain, C. I. Edvard Smith

**Affiliations:** 1 Department of Laboratory Medicine, Clinical Research Center, Karolinska Institutet, Karolinska University Hospital Huddinge, SE-141 86 Huddinge, Sweden; 2 Departamento de Ciencias Básicas, Universidad Industrial de Santander, 680002 Bucaramanga, Colombia; 3 Department of Experimental Medical Science, Lund University, SE-221 84 Lund, Sweden; 4 Department of Hematology, Huai’an First People’s Hospital, Nanjing Medical University, Nanjing, 223300 Jiangsu Republic of China; 5 Department of Medicine Huddinge, Center for Hematology and Regenerative Medicine, Karolinska Institutet, 17177 Stockholm, Sweden; 6 College of Agricultural Engineering Sciences, Salahaddin University-Erbil, 44002 Erbil, Kurdistan Region Iraq; 7 Centre for Rare Diseases, Department of Clinical Genetics, Karolinska University Hospital, SE-171 76 Stockholm, Sweden

**Keywords:** Immunoproliferative disorders, Leukaemia, Cancer therapeutic resistance, Molecularly targeted therapy

## Abstract

Irreversible inhibitors of Bruton tyrosine kinase (BTK), pioneered by ibrutinib, have become breakthrough drugs in the treatment of leukemias and lymphomas. Resistance variants (mutations) occur, but in contrast to those identified for many other tyrosine kinase inhibitors, they affect less frequently the “gatekeeper” residue in the catalytic domain. In this study we carried out variation scanning by creating 11 substitutions at the gatekeeper amino acid, threonine 474 (T474). These variants were subsequently combined with replacement of the cysteine 481 residue to which irreversible inhibitors, such as ibrutinib, acalabrutinib and zanubrutinib, bind. We found that certain double mutants, such as threonine 474 to isoleucine (T474I) or methionine (T474M) combined with catalytically active cysteine 481 to serine (C481S), are insensitive to ≥16-fold the pharmacological serum concentration, and therefore defined as super-resistant to irreversible inhibitors. Conversely, reversible inhibitors showed a variable pattern, from resistance to no resistance, collectively demonstrating the structural constraints for different classes of inhibitors, which may affect their clinical application.

## Introduction

Bruton Tyrosine Kinase (BTK) belongs to the TEC-family of non-receptor tyrosine kinases and is an important component of the B-Cell Receptor (BCR) signaling pathway [1, 2]. BTK is essential for the development and survival of B-cells [3]. Loss-of-function variations in BTK cause X-Linked Agammaglobulinemia [4, 5] due to a block in the B-cell development at the transition from the pro-B to the pre-B cell stage, causing an increased proportion of pro-B and pre-B-I cells and a reduction of all subsequent stages [6, 7]. While aberrant BTK activation and expression have been reported in malignant B-cells [8], it is generally considered that many B-cell tumors are addicted to BTK, since intact BCR-signaling is needed for tumor cells to thrive [9–11]. In a similar way, overexpression and constitutive phosphorylation of BTK lead to the activation of phospholipase C-γ2 (PLCG2), and of extracellular signal-regulated kinase (ERK) and nuclear factor kappa-beta, which promote upregulation of pro-survival signals and migration of chronic lymphocytic leukemia (CLL) cells [12, 13]. Although the need for enhanced BCR-signaling remains elusive, signaling through BTK promotes adhesion and chemotaxis [14, 15].

BTK inhibition is an effective strategy that has revolutionized the treatment of B-cell malignancies [12, 16, 17]. Ibrutinib (Imbruvica^®^) is the most studied BTK inhibitor and the first in this new class approved by the US Food and Drug Administration (FDA) and the European Medicines Agency (EMA) [18]. Resistance to ibrutinib treatment has been attributed to the selection of cells carrying a pathogenic mutant altering BTK or its downstream effector PLCG2 [19]. The most common resistance variation results in a cysteine (C) to serine (S) substitution at position 481, which prevents the covalent binding of ibrutinib to the thiol group located at the ATP-binding site [20, 21]. When this alteration is introduced into the germline of mice, B-cell development remains normal, demonstrating functional interchangeabilty [22]. The BTK variants C481F, C481G, C481R and C481Y are enriched in some CLL patients, but occur at much lower frequency than C481S [21, 23, 24].

Phosphorylation of tyrosines Y551 and Y223 reflects the activation status and catalytic activity of BTK, respectively [25]. Ibrutinib inhibition effectively reduces autophosphorylation of Y223 in wild-type BTK as well as the phosphorylation of PLCG2, but does not impair activity of BTK variants C481S/T [26]. C481S/T variants are resistant to ibrutinib treatment, whereas C481G has only very weak activity upon exposure, and C481F/R/W/Y are catalytically inactive [26].

A new generation of irreversible and reversible BTK inhibitors has been developed to reduce side effects and to overcome resistance toward ibrutinib treatment [27]. Acalabrutinib is a second-generation BTK inhibitor, which covalently binds to C481. It has higher selectivity and reduces the number of adverse effects compared to ibrutinib [28, 29]. Acalabrutinib has been approved by the FDA for the treatment of mantle cell lymphoma (MCL) and CLL/small lymphocytic leukemia [30]. Zanubrutinib is also a more selective, irreversible BTK inhibitor and FDA approved for the treatment of MCL [31]. Zanubrutinib shows potent preclinical activity and minimal off-target effects in patients with Waldenström macroglobulinemia [32, 33].

The gatekeeper residue in BTK is located in the regulatory spine, a conserved structure and key component in the control of the TEC-family kinase domain activity [34]. The gatekeeper residue plays an important role in the access to a deep pocket in the catalytic domain, and activation of an *isolated* kinase domain is independent of its N-terminal portion, as has been demonstrated by threonine to methionine substitution of the gatekeeper residue [35].

Reversible, non-covalent inhibitors are also selective for BTK, and since they do not bind to C481, the inhibition is likely to be at least partially maintained in presence of the C481S variant, in analogy with previous reports [36]. Thus, non-covalent inhibitors have shown high capacity against BTK variants including C481R and T474I/M in in vitro assays [36]. The non-covalent BTK inhibitor fenebrutinib (GDC-0853) was demonstrated to be safe and has been used in phase I studies [37]. Equivalent BTK inhibition was shown for wild-type and the C481S variant as measured by Y223 phosphorylation, when transfected into HEK-293T cells [38]. Viability was reduced and chemokine CCL3 production was decreased in C481S patient-derived clones treated with fenebrutinib in comparison to ibrutinib [38]. Another non-covalent inhibitor, RN486, is under preclinical evaluation and prevents type I and type III hypersensitivity responses, including anti-inflammatory effects in mice with collagen-induced arthritis [39, 40]. CGI-1746, which also binds reversibly to BTK, has been used in rheumatoid arthritis and multiple myeloma mouse models [41].

Acquired mutations at the gatekeeper residue play an important role by causing resistance to many tyrosine protein kinase inhibitors [9]. For example, in chronic myeloid leukemia a T315I replacement in the BCR-ABL fusion causes resistance to the kinase inhibitor imatinib [42]. The anaplastic lymphoma kinase-inhibitor, crizotinib, is affected by the gatekeeper mutation L1196M [43]. In lung cancer, T790M substitution in epidermal growth factor receptor (EGFR) results in resistance to gefitinib, erlotinib, and afatinib. The mechanism of resistance differs from that in BTK, because T790M in EGFR increases the ATP-binding affinity [44]. In contrast, in BTK, the gatekeeper residue T474 is located at the edge of the regulatory spine, which maintains a compact and linear architecture important for BTK activation and function [45].

Mutated gatekeeper residues have been reported in CLL patients resistant to ibrutinib treatment, T474I and T474S were found together with the C481S mutation [46], and a recent study highlighted their importance for resistance to both reversible and irreversible inhibitors as well as the spontaneous occurrence of double-mutants [47]. Here, we evaluate the susceptibility of 16 BTK single and double variants in the gatekeeper residue to both covalent and non-covalent BTK inhibitors.

## Materials and methods

### Generation of plasmids

All plasmids expressing the single substitutions (T474A, T474E, T474F, T474I, T474L, T474M, T474N, T474P, T474Q, T474S, T474V) and double variants (T474A/C481S, T474I/C481S, T474M/C481S, T474M/C481T, and T474S/C481S) were generated on pLX304-Btk (Table 1) and every site-directed mutation was verified by sequencing (Mutagenex Inc. Suwanee, GA, USA).

**Table 1:** Substitutions at T474 and C481.

Substitutions at T474							Substitutions at C481	
Single substitution	Alanine	Asparagine	Isoleucine	Proline	Serine		Serine	Threonine
	(A)	(N)	(I)	(P)	(S)		(S)	(T)
	GCT	AAT	ATT	CCT	TCT			
Two or three substitutions	Fenilalanine	Glutamic acid	Glutamine	Leucine	Methionine	Valine	AGC	ACC
	(F)	(E)	(Q)	(L)	(M)	(V)		
	TTC	GAA	CAG	CTG	ATG	GTG		

### Cell culture and transient transfections

COS-7 and HEK-293T cells were grown in DMEM supplemented with 10% heat-inactivated fetal bovine serum (FBS). The BTK-knock-out, B7.10 cell line, derived from the DT40 (chicken B lymphoma cell line) was kindly provided by Dr T. Kurosaki [48]. B7.10 cells were cultured and maintained in RPMI-1640 medium with 10% FBS, 5% chicken serum, 50 μM 2-mercaptoethanol and penicillin/streptomycin. All cells were grown at 37 °C in a humidified atmosphere with 5% CO_2_.

Plasmids were transiently transfected into COS-7 and HEK-293T cells by using polyethylenimine (PEI) (Polyscience, Inc., Warrington, PA, USA). Electroporation of B7.10 cells was performed by the neon transfection system (Life Technologies, Carlsbad, CA, USA) using a single pulse with 2000 V for 20 milliseconds.

### Inhibitors

All BTK inhibitors were kept at −20 °C and dissolved in dimethyl sulfoxide (DMSO) at 10 mM concentration. For each experiment fresh dilutions were prepared in phosphate buffered saline (Sigma-Aldrich). 36–48 h post transfection, cells were starved under serum-free conditions for 5 h and inhibitors were added during the last hour of starvation. BTK inhibitors were purchased from the following suppliers, ibrutinib and acalabrutinib (Selleckchem, Houston, TX, USA); zanubrutinib (Chemgood, Glen Allen, VA, USA); RN486, CGI-1746 and fenebrutinib (MedChemTronica, Stockholm, Sweden).

### Western blotting

Cells were collected after starvation-inhibition and then activated for 5 min at room temperature. Washout experiments were performed three times in serum-free medium prior to activation. COS-7 and HEK-293T cells were activated with pervanadate [0.02% H_2_O_2_, 1.6% Tyrode’s salt solution (Sigma-Aldrich) and 0.22 mM Na_3_VO_4_ (Sigma-Aldrich)] and FBS (2%), whereas B7.10 cells were activated with H_2_O_2_ (4 mM) and mouse anti-chicken IgM (10 μg/ml, clone M-4, Southern Biotech, Birmingham, AL, USA).

Whole-cell lysate was obtained by using modified RIPA buffer (50 mM Hepes, 120 mM NaCl, 1% NP40, 10% glycerol, and 0.5% sodium deoxycholate) containing a phosphatase inhibitor cocktail (Roche, Basel, Switzerland). Cell lysates were preheated for 5 min at 65 °C with sample buffer (0.2 M sodium carbonate, 0.25 M DL-dithiothreitol, 0.5% glycerol, and 2% sodium dodecyl sulfate). Immunoblotting was performed as previously described [26]. The following antibodies were used for the immunoblotting, polyclonal rabbit anti-BTK and anti-actin (Sigma-Aldrich): mouse anti-BTK (pY551), clone 24a/BTK (Y551) was from BD Biosciences; anti-BTK (pY223) clone EP420Y and polyclonal anti-PLCG2 (pY753) were from Abcam; rabbit anti-PLCG2 polyclonal antibody was from Southern Biotech. Odyssey infrared imaging system was used for scanning after the membranes were incubated with secondary antibodies according to the manufacturer’s protocol (all the secondary antibodies and imaging system were from LI-COR Biosciences GmbH). The signals of total and phosphorylated proteins from duplicate/triplicate or higher number of experiments were quantified by the densitometric program NIH ImageJ 1.52a. β-actin served as internal loading control and the values were normalized to wild-type BTK.

### Computational analyses

BTK-inhibitor co-crystal structures were obtained for ibrutinib (Protein Data Bank id 5p9j) [49], CGI-1746 (5p9g) [49], fenebrutinib (5vfi) [50], RN486 (3ocs, Di Paolo et al. unpublished) and zanubrutinib (6j6m) [51]. The structure for acalabrutinib was obtained from PubChem. Three-dimensional structure for acalabrutinib was docked to the structure with ibrutinib by matching corresponding atoms in the ligands. Docking and visualizations were generated with UCSF Chimera [52]. Side chain substitutions were made with Chimera using in-built side chain rotamer library.

## Results

### Generation of BTK gatekeeper variants

In order to study the efficacy of both covalent and non-covalent BTK inhibitors in the context of resistant CLL cells, we created BTK variants by substituting the gatekeeper T474 residue, known to be associated with drug resistance [46, 53]. Single nucleotide changes in the ACT codon for T474 yield 5 amino acid substitutions T474A/I/N/P/S. T474I and T474S have been found in sub-clones of CLL cells in patients [46]. Variants T474E/F/L/M/Q/V required two or three nucleotide changes. Threonine to methionine substitution in the gatekeeper residue is related to drug resistance e.g., in EGFR [54]. T474E, T474F, T474L, T474Q and T474V replacements change or remove the charge or polarity of the site.

We further investigated the potential influence of gatekeeper residue substitutions when combined with replacement of C481. For that purpose, we generated five double BTK variants: T474A/C481S, T474I/C481S, T474M/C481S, T474M/C481T, and T474S/C481S (Table 1) [26].

### Expression and catalytic activity of BTK gatekeeper variants in COS-7 cells

**Fig. 1:**
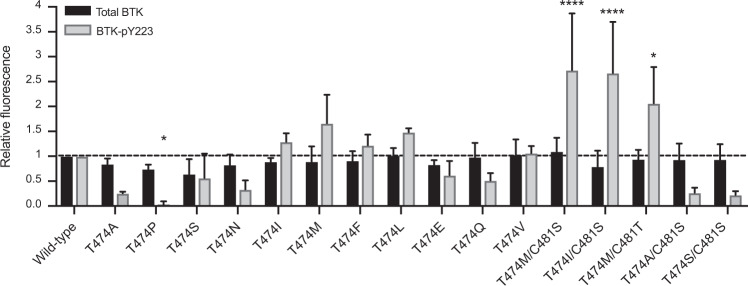
Expression and catalytic activity of BTK gatekeeper variants in COS-7 cells. Thirty-six hours post transfection, the cells were serum starved for 4 h and subsequently activated with serum and pervanadate for 5 min at room temperature. The cell lysates were immunoblotted for total and tyrosine (Y) 223 phosphorylated BTK protein. For densiometric quantification, background signal was subtracted and β-actin utilized as an internal loading control as well as for normalization of total BTK. Values displayed for BTK phosphorylation were normalized both to total protein and wild-type BTK. Four categories were used to quantify the relative BTK activation of the gatekeeper variants compared to wild-type, low (<0.5-fold), intermediate low (0.51–1.0-fold), intermediate high (1.01–1.5-fold) and high (>1.51-fold compared to wild-type). Two-way ANOVA, 95% confidence interval, *p* value ^(^*^)^ < 0.05 and **** < 0.0001.

Constructs with both single and double BTK variants were transfected into COS-7 cells to enable analysis in the absence of endogenous BTK. Expression and activity results are shown in Fig. 1. The expression levels of BTK variants varied ±40% of wild-type.

Phosphorylation of Y223 in the SH3 domain of BTK serves as a measure of BTK catalytic activity [22, 26, 55]. While expressed at similar levels, the variants show activity differences. Single variants T474E/F/I/Q/S/V presented similar Y223 phosphorylation status as wild-type. The catalytic activity of T474A/N/P was substantially reduced, whereas T747L and T747M variants showed increased phosphorylation. Interestingly, the double variants T474I/C481S, T474M/C481S, and T474M/C481T showed significantly, elevated activity, whereas T474A/C481S and T474S/C481S variants exhibited slightly reduced activity.

### Activity of BTK variants in non-lymphoid cells and in B lymphocytes

Further experiments were performed in HEK-293T and B7.10 cells. Similar to COS-7 cells, HEK-293T cells enabled the analysis without the influence of endogenous BTK. The B7.10 cell line is a BTK knock-out subline generated from the parental chicken B lymphoma DT40 [48]. Variants T474I, T474M, and T474S were transfected into these cell lines. Moreover, the double variants T474I/C481S, T474M/C481S, and T474M/C481T, which show higher catalytic activity, were also transfected. When the catalytic activity was evaluated in the three cell lines, COS-7 cells showed the highest increase in phosphorylation at Y223 (Supplementary Fig. 1).

### Gatekeeper variants and their resistance to ibrutinib and acalabrutinib

**Fig. 2:**
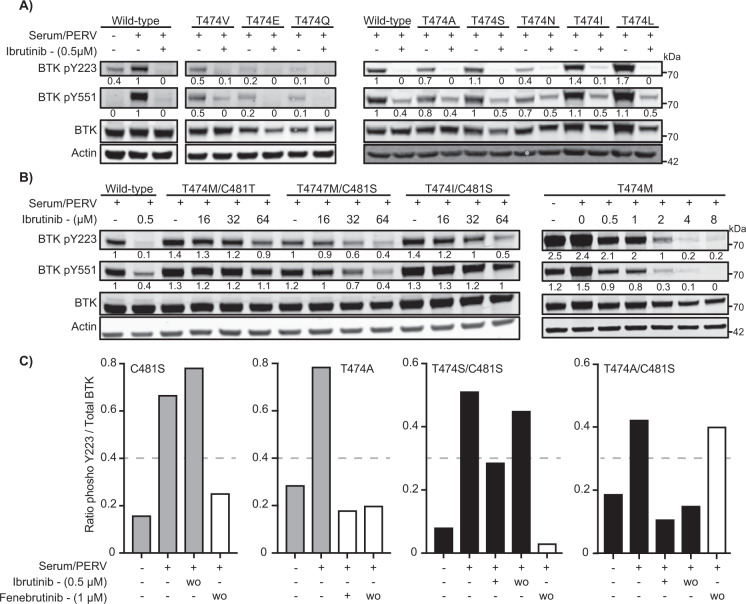
Catalytic activity of BTK variants. COS-7 cells were transfected with wild-type BTK and the enzymatically active BTK gatekeeper variants. Thirty-six hours post transfection, the cells were serum starved for 4 h, BTK inhibitor treated for 1 h and activated for 5 min. BTK protein expression and phosphorylation of Y223 and Y551 were evaluated by immunoblotting. Numbers below bands indicate ratio of phosphorylated protein to total protein as obtained by densiometric quantification with background signal subtracted. β-actin was utilized as an internal loading control and values displayed for BTK phosphorylation were normalized to wild-type. **A** Gatekeeper variants inhibited at 0.5 μM of ibrutinib. **B** Ibrutinib-resistant variants requiring higher ibrutinib concentration. **C** Ratio of Y223 phosphorylated protein over total protein (as quantified by densiometric analysis) from C481S, T474A, T474S/C481S, and T474A/C481S variants. Washout (wo) was performed three times in serum-free medium prior to activation.

To evaluate the efficacy of ibrutinib to block BTK phosphorylation in the gatekeeper variants, we initially exposed transfected COS-7 and HEK-293T cells to ibrutinib in different concentrations. First, the inhibition of BTK phosphorylation at Y223 was analyzed in COS-7 cells using 0.5 μM of ibrutinib, which is the pharmacological concentration obtained in serum of treated patients [56]. Ibrutinib inhibited almost completely Y223 phosphorylation, whereas Y551 phosphorylation, which is dependent on other kinases, was only partially affected (Fig. 2), similar to previous observations [26].

The BTK variants sensitive to ibrutinib were T474A/E/I/L/N/Q/S/V, and unexpectedly also T474A/C481S and T474S/C481S (Fig. 2A and C). While T474S/C481S was partially sensitive, T474A/C481S was fully sensitive at 0.5 μM concentration and resisted three washouts. As expected, the T474M variant was insensitive to ibrutinib at 0.5 μM, whereas the resistance was reduced at 2 μM and lost at a concentration of 4 μM. The double mutants, T474I/C481S, T474M/C481S, and T474M/C481T showed an unexpected super-resistance (defined as ≥16-fold the pharmacological serum concentration) to ibrutinib, for which BTK activity was not robustly blocked even when increased 120-fold (64 μM) over the physiological concentration (Fig. 2B).

The potential effect of the single methionine, isoleucine or leucine substitutions in the gatekeeper residue on ibrutinib binding to BTK was also assessed by washout experiments. Our results show that both T474S/I, being sensitive to 0.5 μM and T474M being only sensitive at 4 μM, variants behave as wild-type with regard to washouts, indicating that these gatekeeper substitutions do not abrogate ibrutinib covalent binding (Supplementary Fig. 2).

Experiments in HEK-293T cells confirmed the results obtained in COS-7 cells. The outcome for T474I/M/S, T474I/C481S, and T474M/C481S was similar to that in COS-7 cells. An exception, for which we have no explanation, was T474M/C481T, whose phosphorylation was essentially completely blocked by 64 μM of ibrutinib in HEK-293T, but was less affected in COS-7 cells (Fig. 2 and Supplementary Fig. 3).

The effect of the variants on the second-generation covalent BTK inhibitor, acalabrutinib, was also tested. We transfected variants T474I/M/S, T474I/C481S, and T474M/C481S into COS-7 cells and exposed the cells to a series of dilutions of acalabrutinib, from 1.5 μM to 96 μM. Similar to ibrutinib, acalabrutinib inhibition is affected by substitutions at both C481 and T474. A concentration of 12 μM of acalabrutinib was needed to block at least 70% of the Y223 phosphorylation in T474M, and the super-resistance was maintained in the double variants, as in the case of ibrutinib treatment. The catalytic activity was not completely blocked even at 96 μM of acalabrutinib (Supplementary Fig. 4).

Zanubrutinib was studied in COS-7 cells transfected with T474I/C481S and T474M/C481S variants. Zanubrutinib is more selective for BTK and has fewer off-targets than ibrutinib [51]. Zanubrutinib binds covalently to C481, and similar to ibrutinib and acalabrutinib, it could overcome the double replacements, measured as phosphorylation at Y223, only at very high concentration (64 μM) (Supplementary Fig. 5). Collectively, this set of data demonstrates that all the investigated irreversible inhibitors were subject to the same pattern of super-resistance, when both C481 and T474 were replaced.

### Non-covalent inhibitors as treatment for BTK variants resistant to ibrutinib

**Fig. 3:**
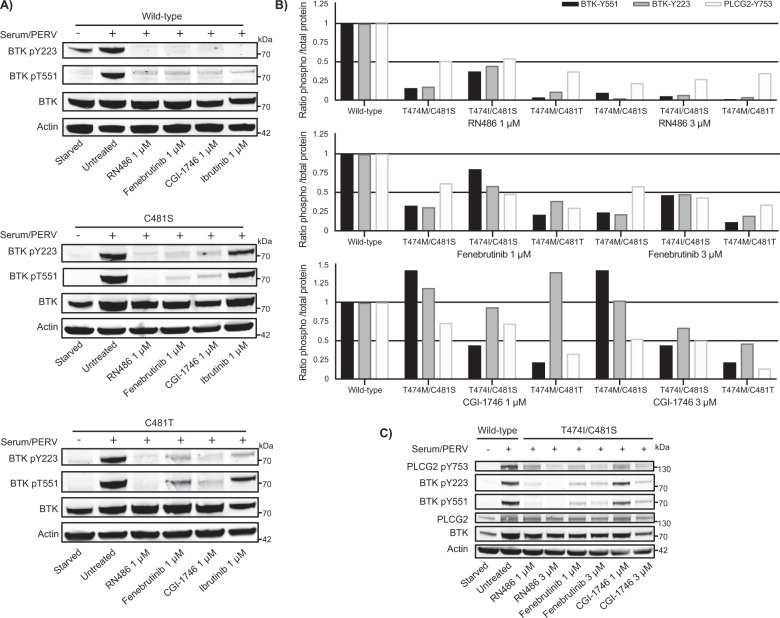
BTK and PLCG2 inhibition by RN486, fenebrutinib and CGI-1746. COS-7 cells were transfected with wild-type BTK and ibrutinib-resistant gatekeeper variants. Thirty-six hours post transfection, the cells were serum starved for 4 h and treated with the BTK inhibitor for 1 h. Activation was performed for 5 min at room temperature using serum and pervanadate. Total (BTK and PLCG2) and phosphorylated protein sites (Y223 and Y551 for BTK; Y753 for PLCG2) were measured using immunoblotting. **A** Ibrutinib, RN486, fenebrutinib and CGI-1746 were tested in transfected cells with wild-type BTK and resistant variants C481S and C481T. **B** Non-covalent BTK inhibitors in COS-7 cells transfected with the resistant variants T474M/C481T, T474I/C481S, and T474M/C481S. Ratio of phosphorylated protein over total protein (as quantified by densiometric analysis). **C** Representative western-blot for T474I/C481S. Western-blots for T474M/C481S and T474M/C481T are included in Supplementary Fig. 6.

Three commercially available, non-covalent BTK inhibitors RN486, fenebrutinib and CGI-1746 were tested in COS-7 cells. Following transfection with either wild-type or variants of BTK the cells were treated with 1 or 3 μM of the compounds. Similar to wild-type BTK (Fig. 3A, top), 1 μM of non-covalent inhibitors was sufficient to block phosphorylation of BTK (Y223 and Y551) in both C481S and C481T variants (Fig. 3A, middle and bottom).

Owing to that the T747A/C481S and T747S/C481S variants unexpectedly were sensitive and partially sensitive to ibrutinib, respectively (Fig. 2C), we were also interested to see how the variants behave when they are used in the presence of non-covalent inhibitors. We tested the effect of fenebrutinib and this non-covalent inhibitor, as anticipated, inhibited the single mutants, C481S and T474A, and also the double T474S/C481S even after washout. However, surprisingly, the T474A/C481S variant was insensitive to fenebrutinib, providing an example of a resistance mutant for this inhibitor that potentially could occur in tumor patients.

**Fig. 4:**
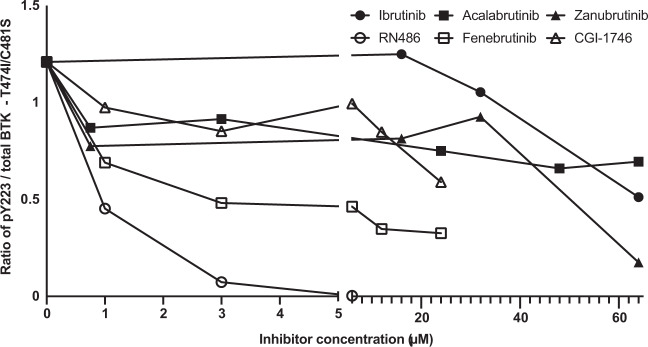
Comparison of the BTK phosphorylation activity blocked by covalent and non-covalent BTK inhibitors in the double variant T474I/C481S. COS-7 cells were transfected with T474I/C481S variant, serum starved and treated with the covalent and non-covalent BTK inhibitors. Activation was performed using serum and pervanadate. Total BTK and the amount of BTK phosphorylated at Y223 were measured using immunoblotting. Ratio of phosphorylated protein over total protein (as quantified by densiometric analysis) are displayed.

In all the ibrutinib super-resistant double-variants, 1 μM RN486 blocked >50% of the BTK activity and ≥50% phosphorylation of the downstream target PLCG2 (Y753), and almost complete BTK inhibition was obtained at 3 μM (Fig. 3B, top and Supplementary Fig. 6). Apart from T474A/C481S, as mentioned, Fenebrutinib inhibited >50% of BTK and PLCG2 phosphorylation in double variants at 1 μM concentration with the exception of T474I/C481S for BTK and T474M/C481S for PLCG2 (Fig. 3B, middle and Supplementary Fig. 6). Fenebrutinib at 3 μM inhibited >50% of the activity of the T474M/C481T variant (Fig. 3B, middle and Supplementary Fig. 6). CGI-1746 did not block BTK activity in most of the double variants at a concentration of 1 or 3 μM (Fig. 3B, bottom and Supplementary Fig. 6). In order to test the possibility of total block of BTK phosphorylation with fenebrutinib or CGI-1746, the concentration of the inhibitors was gradually increased to 6, 12 and 24 μM. The T474I/C481S variant was chosen for this experiment, since both substitutions have been reported in ibrutinib-resistant patients [46]. BTK phosphorylation activity was only partially blocked even when elevated concentrations of fenebrutinib or CGI-1746 were used (Fig. 4). Our results suggest that the tested non-covalent BTK inhibitors could be further examined for the treatment of C481S and C481T variants and that RN486 would offer the best treatment for the double variants T474I/C481S, T474M/C481S and T474M/C481T.

### Bioinformatic and structural analysis of affinity effects due to variants

The effects of the variants were examined utilizing three-dimensional structures. Coordinates were available for five of the complexes, in the case of acalabrutinib the inhibitor structure was downloaded and docked based on the matching atoms in ibrutinib.

**Fig. 5:**
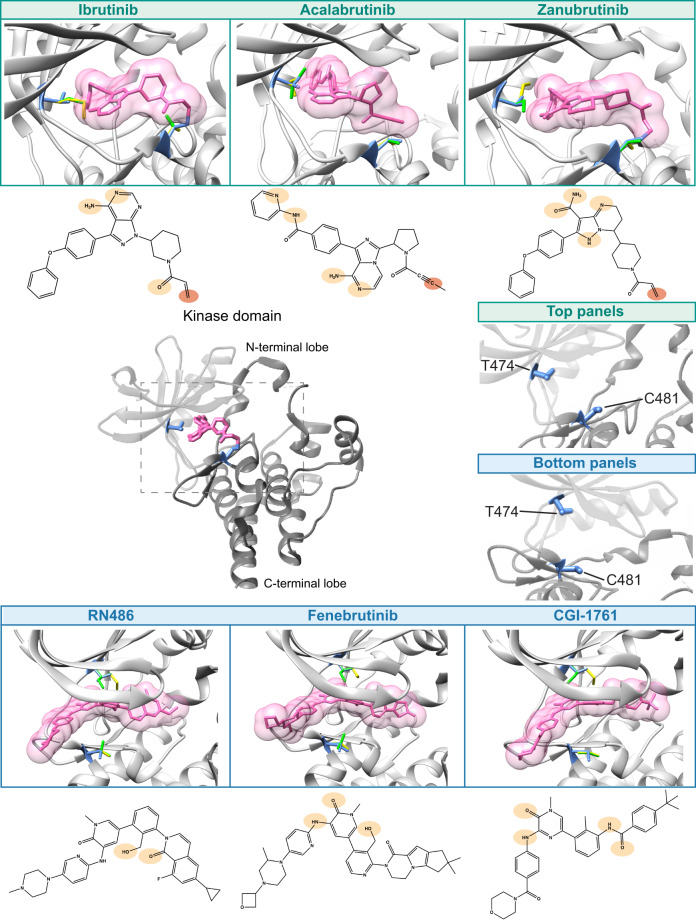
Binding of covalent and non-covalent inhibitors to BTK and effects of amino acid substitutions. The entire kinase domain-ibrutinib complex structure is in the left-center with ibrutinib in the catalytic site. The N-terminal lobe is in light gray and the C-terminal lobe in dark gray. Side chains are shown for threonine (T) 474, top, and cysteine (C) 481, below, in blue. Top row shows the chemical structures and indicates binding of covalent inhibitors and the bottom row of non-covalent inhibitors. Original residues at positions 474 and 481, T and C, respectively, are in blue. Substitutions at 474 methionine (yellow) and isoleucine (green), at 481 serine (yellow) and threonine (green). For the panels 474 and 481 are indicated in the middle. Ovals in light and dark orange indicate the sites of formation of hydrogen bonds and the covalent bond to cysteine, respectively [36, 41, 47, 50, 51, 60].

**Fig. 6:**
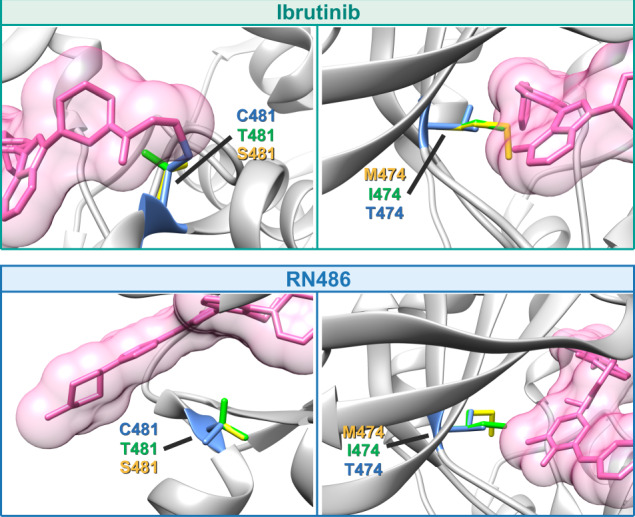
Structural explanations for substitutions. Ibrutinib, top, and RN-484, bottom, are used as examples for covalent and non-covalent inhibitors, respectively. Substitutions of C481 prevent covalent binding of inhibitors, and the side chains can affect the positioning of the inhibitor. Methionine and isoleucine replacements at gatekeeper 474 collide with the covalent inhibitors and affect binding affinity. Non-covalent inhibitors have different binding mode and side chains at 481 are further away from the inhibitor. The gatekeeper residue is important for binding of these inhibitors, as well.

BTK binding of the inhibitors was based on experimental three-dimensional structures, except for acalabrutinib that was modeled in the closed conformation, as the binding is only relevant in it (Fig. 5). Four variants were substituted in the structures (T474M/I and C481S/T). The effects of the substitutions can be explained based on the structure. C481 in the wild-type forms a covalent bond with the inhibitor, but not in the substitutions (Fig. 5; Fig. 6 left). Further, amino acid alterations can collide with the inhibitor. Combined effects create weaker binding of the inhibitors to the variants. Amino acid changes T474M and T474I shorten the side chain in comparison to the wild-type threonine and thus cannot retain the mode of binding (Fig. 5; Fig. 6 right). Note that the side chains are flexible and have several favorable rotamers; however, their capability to bind is reduced and thus higher inhibitor concentrations are expected for activity, consequently leading to super-resistance.

The binding mode of the non-covalent inhibitors is very different in comparison to the covalent inhibitors. The gatekeeper residue T474 is important for binding of both types of inhibitors. Super-resistance in double variants emerges because binding interactions are lost or modified at positions important for affinity and/or specificity.

## Discussion

**Fig. 7:**
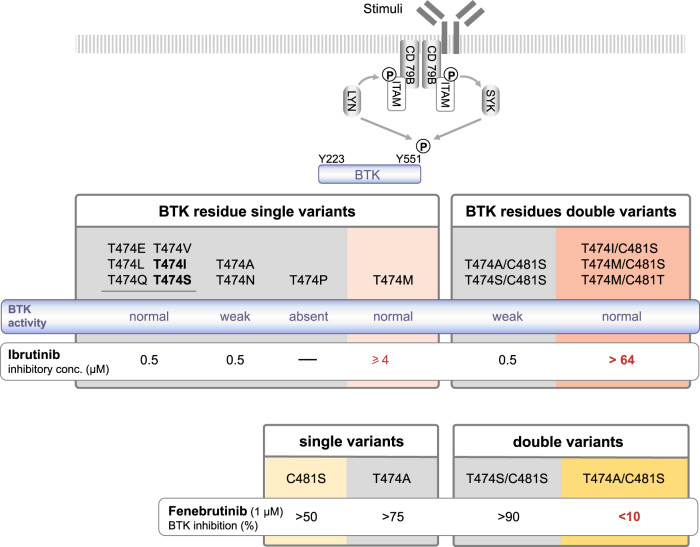
Effects of ibrutinib and fenebrutinib on BTK activity in single and double variants. (top panel) Schematic representation of the BCR-BTK signaling pathway and the (middle panel) identification of ibrutinib super-resistant double mutants. Gatekeeper T474P mutant is not phosphorylated on Y551 and hence evaluation of pY223 cannot be made. Variants in bold represent mutations found in treated CLL patients. *(bottom panel) Selected findings regarding fenebrutinib sensitivity or resistance*. Other covalent BTK inhibitors acalabrutinib and zanubrutinib were also tested at very high concentration and super-resistance was confirmed (Supplementary Figs. 4 and 5). Fenebrutinib was also tested for C481T, T474M/C481S, T474I/C481S and T474M/C481T, see Fig. 3. While other covalent inhibitors are not mentioned in the figure, they behave similar to ibrutinib.

We here demonstrate how simultaneous substitution of the C481 residue, to which irreversible BTK inhibitors tether, and of the gatekeeper amino acid, T474, result in super-resistance to three clinically approved BTK inhibitors (summarized in Fig. 7). These findings also provide insight into the binding mode of both irreversible and reversible inhibitors.

BTK inhibitors are highly efficient in the treatment of CLL and in a group of other B-cell malignancies. Covalent inhibitors ibrutinib, acalabrutinib, and zanubrutinib are approved for clinical use and the non-covalent inhibitor fenebrutinib has been tested in phase 1 and 2 clinical trials [37, 38]. Non-covalent BTK inhibitors are needed because ~60% of ibrutinib long-term treated patients obtain resistant sequence variants, mainly in BTK, but also in PLCG2 [21, 24, 53]. The most frequently mutated site is C481 and the most common substitution is to serine [21, 24], but other substitutions may occasionally predominate [21].

Non-covalent BTK inhibitors provide promising treatment options for patients developing drug resistance [36]. Reduction of the inhibitory capacity was previously reported for spebrutinib and GS-4059 in C481S, C481R, T474I, and T474M variants [36]. The poorest inhibition of spebrutinib and of GS-4059 was obtained in the C481S variant and in the T474I or T474M gatekeeper mutants, respectively [36].

Non-covalent inhibitors have an orthogonal binding mode and occupy the kinase domain H3 pocket forming several hydrogen bonds [36, 47, 50]. Inhibitors such as RN486, bind to BTK using a network of three hydrogen bonds to the kinase-invariant residues K430 and G414 and to T474 (Fig. 5) [47].

Gatekeeper variations have been detected in treated CLL patients with a frequency of 4% and in the presence of the resistant C481S variation [46]. The role of the gatekeeper variations in BTK is not well understood, but since tumor cells carrying them increase in numbers, they act as resistance drivers. A gatekeeper variant could affect the binding of both covalent and non-covalent BTK inhibitors. Substitutions T474I or T474M introduce longer side chains that could sterically interfere the binding of covalent inhibitors and might disrupt the hydrogen bonds needed for the binding of non-covalent inhibitors [36, 47]. In a systematic BTK mutagenesis screen, the importance of the T474 variants was reported for non-covalent inhibitors binding [47]. Interestingly, the authors observed co-occurrence of gatekeeper and kinase domain variants (L512M, E513G, F517L, L547P) in *cis*. Although two variations affecting the same allele might be anticipated to be very rare, they are relatively common in breast cancer patients with alterations to the catalytic subunit of the phosphoinositide 3-kinase alpha (PI3Kα) complex, 95% carrying the double-variant E545K/E726K [57].

Upon substitution of the gatekeeper residue, we found that most, but not all, variants are expressed at normal levels and are catalytically active. BTK was inhibited by ibrutinib in all the single variants with the exception of T474M, which does not explain the enrichment of T474I clones in a subset of treated patients [46]. Variant activities were blocked by 0.5 μM of ibrutinib, which is equivalent to the peak inhibitor concentration in plasma of treated patients [56]. To inhibit the T474M variant, 4 μM of ibrutinib was needed. These results confirm that methionine substitution affects the potency of covalent inhibitors even when C481 is intact in BTK [36].

We generated five double variants to investigate effects on inhibitor binding. T474I/C481S, T474M/C481S and T474M/C481T variants are super-resistant to ibrutinib, while T474A/C481S and T474S/C481S are sensitive and partially sensitive, respectively, at the clinically relevant 0.5 μM concentration. We hypothesize that super-resistance appears when ibrutinib cannot bind covalently to C481S, it acts instead as non-covalent inhibitor and forms hydrogen bonds with E475 and M477 similar to in the wild-type [36]. The replacements at position 474, either by methionine or isoleucine, could alter the binding site and weaken affinity to ibrutinib even at very high concentrations (Fig. 6). The super-resistant variants to ibrutinib T474M/C481S, T474I/C481S and T474M/C481T are also unsusceptible to acalabrutinib and zanubrutinib.

The sensitivity of T474A/C481S and T474S/C481S variants to ibrutinib is compatible with explanation that substitutions carrying smaller side chains are less likely to clash with other residues. In T474S/C481S the polar character of the side chain is also retained. The single variants T474A and T474S showed slightly reduced enzymatic activity, as previously reported for T474A [35], but since T474S-carrying CLL cells are enriched in ibrutinib-treated patients [46], albeit rarely, this means that the activity is sufficient for tumor cells to thrive. Yet, it was unexpected that the double substitutions T474S/C481S and T474A/C481S were partially, and fully sensitive, respectively, to ibrutinib at pharmacological concentration. The T474A and T474S gatekeeper replacements alter the binding pocket making it wider, however the serine variant could retain hydrogen binding to ibrutinib. In the case of alanine, we can hypothesize that a water molecule bound relatively stable could compensate for the missing side chain interactions, alternatively the binding site is changed along with adjustment in the angle between the lobes. This resistance mechanism is not unprecedented, since better fit for ATP has been reported in non-small-cell lung cancer patients treated with tyrosine kinase inhibitors. Variations affecting the gatekeeper threonine residue in the EGFR were identified as the underlying mechanism [9].

The non-covalent BTK inhibitors RN486 and CGI-1746 are highly selective for BTK in in vitro and in vivo models, implying a potential for treatment of patients with C481S resistant variations [36, 40, 41, 58]. Fenebrutinib demonstrated clinical activity in a phase 1 study and inhibitory capacity of C481S variant in preclinical data [37, 38]. Two other non-covalent BTK inhibitors, vecabrutinib (SNS-062), ARQ 531 and LOXO-305 were also shown to be effective against the C481S variant, as recently reviewed [59]. Our results for C481S and C481T variants confirm that all the three non-covalent BTK inhibitors could be further examined as treatment for ibrutinib-resistant patients. Only RN486 showed inhibitory capacity at a clinically relevant concentration against T474M/C481S, T474I/C481S, and T474M/C481T, and may provide the most potent treatment option.

## Supplementary information

Supplementary data
