# Supplementary material for: BTK gatekeeper residue variation combined with cysteine 481 substitution causes super-resistance to irreversible inhibitors acalabrutinib, ibrutinib and zanubrutinib
Source: Leukemia. 2021 Feb 1;35(5):1317–29. doi: 10.1038/s41375-021-01123-6 (PMC8102192; doi:10.1038/s41375-021-01123-6)
Supplement: Supplementary file 1 — Supplementary data [file 41375_2021_1123_MOESM1_ESM.pdf]

Supplementary data

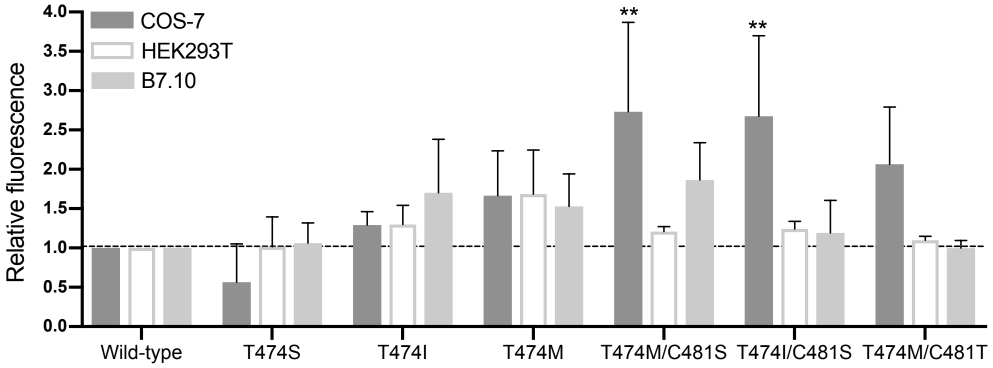

Supplementary Figure 1. **Phosphorylation levels at Y223 in cell lines COS-7, HEK-293T and B7.10**, **which were transfected with wild-type BTK or the indicated BTK variants**. Lysates from previously starved and activated cells were immunoblotted for total and phosphorylated BTK protein. For densiometric quantification, background signal was subtracted, β-actin utilized as an internal loading control and values displayed were normalized to total protein. Two-way ANOVA, 95% confidence interval, *p*-value **<0.002.

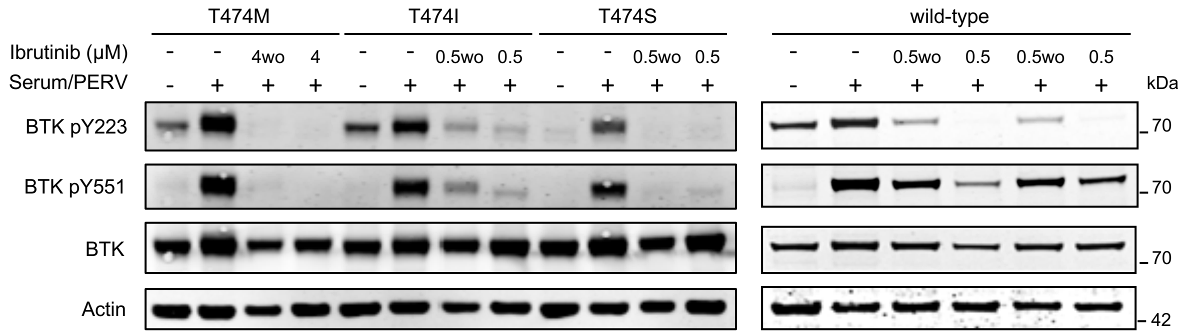

Supplementary Figure 2. **Ibrutinib washout experiments for wild-type and T474M, T474I and T474S BTK variants transfected in COS-7 cells.** COS-7 cells were transfected with the wild-type and BTK variants T474M/I/S. Lysates from previously starved, BTK inhibitor treated and activated cells were immunoblotted for total and phosphorylated BTK protein. In samples indicated as wo (washout), washout was performed three times in serum-free medium prior to activation.

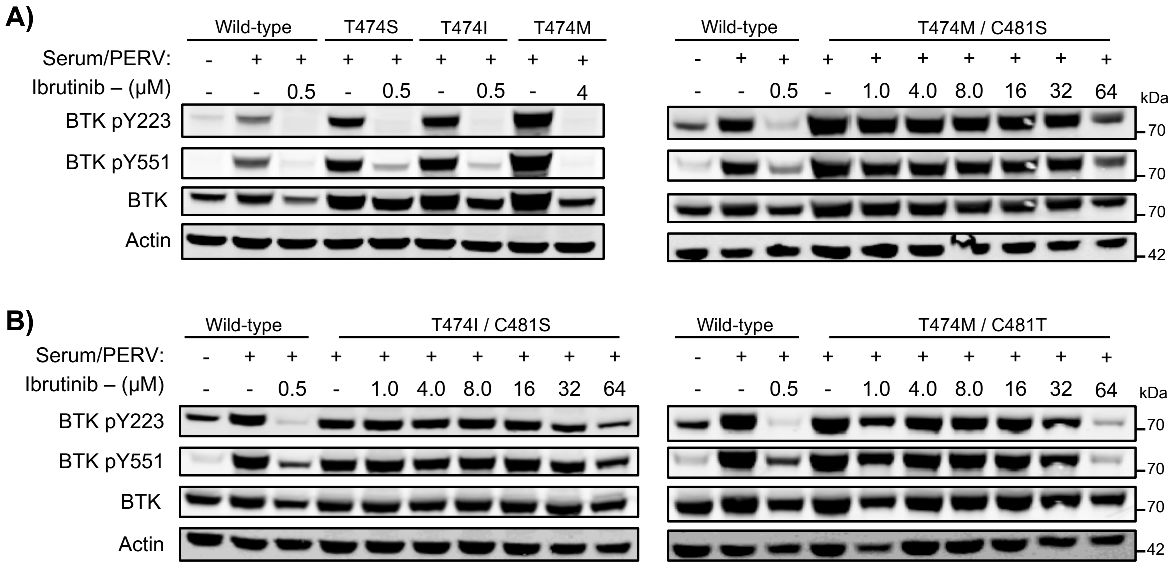

Supplementary Figure 3. **Inhibition of BTK in HEK293 cells with variants T474I and T474S**, **which have emerged in sub-clones of CLL patients and the identified ibrutinib-resistant double variants.** Lysates from previously starved, ibrutinib treated and activated cells were immunoblotted for total and phosphorylated BTK protein.

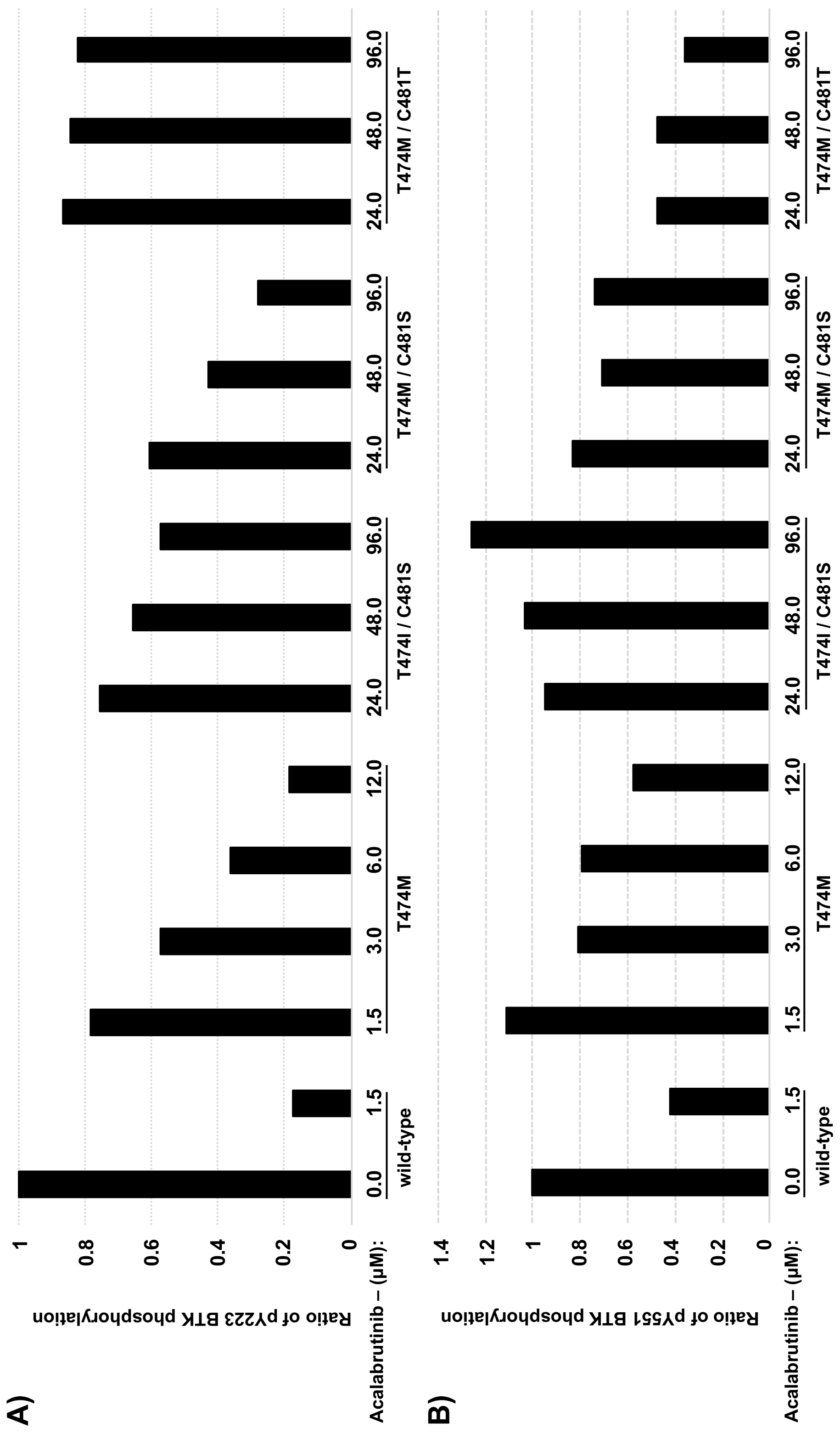

Supplementary Figure 4. **Acalabrutinib inhibition of BTK in ibrutinib-resistant variants measured as Y223 phosphorylation.** Thirty-six hours post transfection, the cells were serum starved and treated with acalabrutinib for 1 h. Activation was performed for 5 min at room temperature using serum and pervanadate.

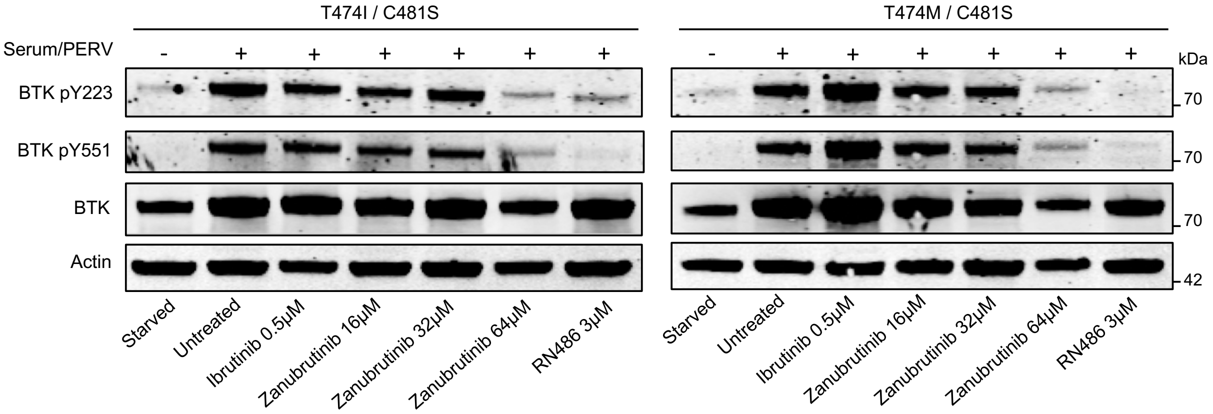

Supplementary Figure 5. **Inhibition of T474I/C481S and T474M/C481S BTK variants with zanubrutinib in COS-7 cells.** COS-7 cells were transfected with the BTK variants T474I/C481S and T474M/C481S. Lysates from previously starved, BTK inhibitor treated and activated cells were immunoblotted for total and phosphorylated BTK protein.

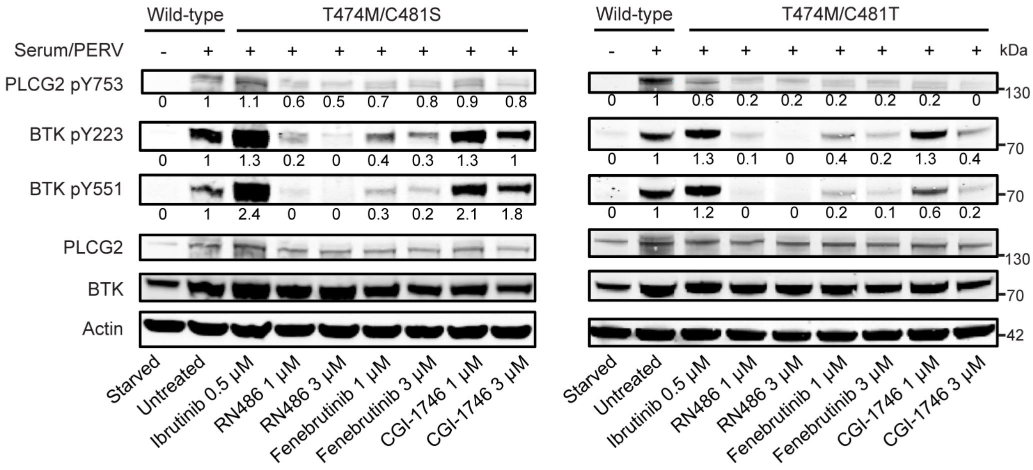

Supplementary Figure 6. **BTK and PLCG2 inhibition by RN486, fenebrutinib and CGI-1746 in COS-7 cells transfected with T474M/C481S and T474M/C481T.** Corresponding western-blot quantification is shown as bars in Fig. 3b. Numbers below bands indicate the relative ratio of phosphorylated protein to total protein obtained by densiometric quantification with ratio for wild-type BTK set as 1. For densiometric quantification, background signal was subtracted, β-actin was utilized as an internal loading control and values displayed for BTK and PLCG2 phosphorylation were normalized to wild-type.
